# Circular RNA circ_0026218 Suppressed Atherosclerosis Progression via miR-338-3p/SIRT6 Axis

**DOI:** 10.1155/2023/5647758

**Published:** 2023-01-24

**Authors:** Liang Yang, Wei Chen, Bin Li, Yvbao Hu, Hua Lu, Pan Zhang, Huayun Yang, Mochen Zhang, Diguang Pan

**Affiliations:** Heart Center, Guilin People's Hospital, Guilin, 541002 Guangxi, China

## Abstract

**Background:**

Multiple circular RNAs (circRNAs) are implicated in atherosclerosis (AS) pathogenesis. In fact, how circRNA 0026218 (circ_0026218) functions in AS remains unknown, and thus the functions and mechanisms of circ_0026218 in the injury of vascular endothelial cells are to be investigated.

**Methods:**

Microarray analysis was employed to screen out differentially expressed circRNAs in AS. A cell model was mimicked by treating Human umbilical vein endothelial cells (HUVECs) with oxidized low-density lipoprotein (ox-LDL). circ_0026218, microRNA-338-3p (miR-338-3p) and silent information regulator 6 (SIRT6) expressions in HUVECs with ox-LDL treatment were probed by qRT-PCR. The cell proliferative capabilities were exposed by CCK-8 assay. The contents of interleukin 6 (IL-6), interleukin 1*β* (IL-1*β*), and tumor necrosis factor *α* (TNF-*α*) were measured by ELISA. Oxidative stress kits were utilized to detect the levels of reactive oxygen species (ROS), superoxide dismutase (SOD), and malondialdehyde (MDA). Flow cytometry was adopted to analyze the level of apoptosis of HUVECs. Dual-luciferase reporter gene assay and RIP assay were leveraged to expose the interplay between miR-338-3p and circ_0026218 or SIRT6 3′-UTR, respectively. In addition, the impacts of circ_0026216 and miR-338-3p on SIRT6 protein expressions were subjected to Western blot.

**Results:**

circ_0026218 was greatly depleted in ox-LDL-stimulated HUVECs. circ_0026218 overexpression promoted viability of HUVECs *in vitro* and inhibited inflammatory response, oxidative stress, and apoptosis. circ_0026218 could adsorb miR-338-3p and positively modulated SIRT6 expressions via sponging miR-338-3p. Upregulation of this miRNA reversed the influence of circ_0026218 overexpression on ox-LDL-caused injury and apoptosis of HUVECs.

**Conclusion:**

Collectively, circ_0026218 upregulates SIRT6 expression through decoying miR-338-3p, thereby inhibiting ox-LDL-initiated injury of HUVECs. circ_0026218 is involved in the pathogenesis of AS.

## 1. Introduction

Atherosclerosis (AS) appears as the pathological basis of diverse cardiovascular and cerebrovascular diseases such as cerebral infarction, coronary artery disorder, and peripheral vascular disease [1]. Endothelial cells have important functions in maintaining vascular homeostasis; their dysfunction is an early biological event of AS [2]. Various adverse stimuli (e.g., stress, inflammation, and hypoxia) can lead to endothelial cell injury and apoptosis in AS [3, 4]. Oxidized low-density lipoprotein (ox-LDL) is a vital pathological part of AS. ox-LDL causes endothelial cell dysfunction and apoptosis, which triggers the formation of AS plaques [5]. Therefore, elucidation of ox-LDL-initiated endothelial cell dysfunction is pivotal for curing AS.

Circular RNA (circRNA) is a category of endogenous noncoding RNA transcripts with a covalently closed loop structure [6]. Reportedly, circRNAs feature prominently in multiple cardiovascular diseases [7]. For example, circ_0004543 is highly expressed in ox-LDL-initiated human umbilical vein endothelial cells (HUVECs), and circ_0004543 depletion increases the growth of ox-LDL-caused HUVECs [8]. circ_0065149 restrains ox-LDL-caused apoptosis and inflammation of HUVECs via targeting miR-330-5p [9]. circTM7SF3 promotes ox-LDL-mediated injury of macrophages by sponging miR-206 to raise ASPH expression [10]. In this work, circRNA_0026218 (circ_0026218) was abnormally depleted in phorbol 12-myristate 13-acetate-induced macrophages by analyzing circRNA microarrays (GSE107522). Macrophages' differentiation into foam cells is the key biological process in the pathogenesis of AS, and this result suggests that circ_0026216 participates in AS development. However, how circ_0026218 functions in the injury of endothelial cell is not clear.

MicroRNAs (miRNAs/miRs) are conceptually a category of small noncoding RNA transcripts of 21-25 nt that modulate gene expressions posttranscriptionally by restraining the translation of target mRNAs [11]. As reported, miRNAs feature prominently in the pathological processes of AS, lipoprotein metabolism, endothelial cell viability and apoptosis, and immune response included [12]. MicroRNA-338-3p (miR-338-3p) impairs the multiplication and interferes with the progression of gastric cancer [13], and glioblastoma [14]. Reportedly, in AS, miR-338-3p alleviates endothelial cell injury caused by ox-LDL via targeting BAMBI and initiating the TGF-*β*/Smad pathway [15]. Nonetheless, the potential mechanism that miR-338-3p mediates AS development has not been elucidated. Silent information regulator 6 (SIRT6), part of the sirtuin family, is a histone/nonhistone deacetylase that utilizes NAD^+^ as a cofactor [16]. As reported, SIRT6 can prevent vascular smooth muscle cells from senescence and inhibit the development of AS [17]. Moreover, SIRT6 overexpression attenuates microcholesterol crystal-induced endothelial dysfunction [18]. Nevertheless, the mechanisms of SIRT6 dysregulation in endothelial cells need to be further elucidated.

This work is aimed at investigating the expression features and biological function of circ_0026218 in endothelial cells during the development of AS, and exploring its relations with miR-338-3p and SIRT6.

## 2. Materials and Methods

### 2.1. Bioinformatics Analysis

The microarray dataset GSE107522 was available from the Gene Expression Omnibus database, and microarray data analysis was accomplished as described previously [19]. Briefly, the interactive web tool GEO2R was leveraged to analyze the expression profile of circRNAs in macrophages treated with 100 nM phorbol 12-myristate 13-acetate, and stimulated with or without ox-LDL (50 mg/mL). circRNAs with *P* < 0.05 and log_2_^(foldchange)^ > 1 or < -1 were reflective of significant differences.

### 2.2. Cell Culture and ox-LDL Treatment

HUVECs were available from the China Center for Type Culture Collection (Wuhan, China). Human embryonic kidney (HEK)-293 cells were from American Type Culture Collection (Manassas, VA, USA). They were notably cultured in Dulbecco's modified Eagle's medium (Invitrogen, Carlsbad, CA, USA) with 10% fetal bovine serum (FBS) (Gibco, Rockville, MD, USA), 100 *μ*g/mL of streptomycin, and 100 U/mL of penicillin (Gibco) at 37°C in 5% CO_2_. HUVECs were followingly treated with discrepant concentrations (0, 50, 75, and 100 *μ*g/mL) of ox-LDL (Sigma-Aldrich, St. Louis, MO, USA) at varied period points (0, 24, 48, and 72 h) when they were in the logarithmic growth phase, as described previously [20].

### 2.3. Cell Transfection

circ_0026218 overexpression plasmid (circ_0026218-oe), miR-338-3p mimics, and their negative control (vector, miR-con) were from GenePharma (Shanghai, China). Specifically, the vectors and oligonucleotides (50 nM) were immediately transfected into HUVECs by the Lipofectamine®2000 reagent (Invitrogen).

### 2.4. Quantitative Real-Time Polymerase Chain Reaction (qRT-PCR)

Total RNA was extracted by TRIzol reagent (Thermo Fisher Scientific, Waltham, MA, USA) and reversely transcribed into complementary DNA (cDNA) by the TaqMan miRNA Reverse Transcription Kit (Applied Biosystems, Foster City, CA, USA). Notably, qRT-PCR was accomplished on a 7900HT Fast Real-Time PCR System (Applied Biosystems) using SYBR™ Green PCR Master Mix (Applied Biosystems). Relative expressions were rated by 2^-*ΔΔ*Ct^ method. U6 (for miR-338-3p) and GAPDH (for circ_0026218 or SIRT6) worked as internal controls. The primer sequences: circ_0026218 Forward: 5′-CAAGTATCAGCGGCTCTGTG-3′, reverse: 5′-TGTCCTCGATGCCAATACAG-3′; SIRT6, forward: 5′-CCGGAGGAGCTGGAGCGGAAG-3′, reverse: 5′-CGTGGGCCGCGCGCTCTCAAAG-3′; GAPDH, forward: 5′-TGGATTTGGACGCATTGGTC-3′, reverse: 5′-TTTGCACTGGTACGTGTTGAT-3′; miR-338-3p, forward: 5′-GGGGTACCGAATCTTCCCAGTAGGCG-3′, reverse: 5′-TTGCGGCCGCAAAGGAGAAGGGCCAAAC-3′; U6, forward: 5′-CTCGCTTCGGCAGCACA-3′, reverse: 5′-AACG CTTCACGAATTTGCGT-3′.

### 2.5. Ribonuclease (RNase) R Assay

2 mg of total RNA samples were incubated for 15 min at 37°C in the presence or absence of 3 U/mg RNase R (Epicenter Biotechnologies, Madison, WI, USA). Next circ_0026218 and GAPDH expressions were detected by qRT-PCR.

### 2.6. Subcellular Distribution Assay

Nuclear and cytoplasmic RNAs were extracted from HUVECs using NE-PER Nuclear and Cytoplasmic Extraction Reagents (Thermo Fisher Scientific). circ_0026218, U6, and GAPDH were then detected by qRT-PCR, with U6 and GAPDH as nuclear and cytoplasmic controls, respectively.

### 2.7. Cell Viability Assay

HUVCEs were inoculated into 96-well plates (about 10,000 per well). Then, 10 *μ*L of cell counting kit-8 (CCK-8) solution (MedChem Express, Monmouth Junction, NJ, USA) was dripped into each well and subsequently incubated for 3 h at 37°C. Thereafter, the value of OD_450 nm_ was measured by a microplate reader (Bio-Rad Labs, Richmond, CA, USA).

### 2.8. Enzyme-Linked Immunosorbent Assay (ELISA)

The supernatant of each group of HUVECs was gathered. Besides, interleukin (IL)-1*β*, IL-6, and tumor necrosis factor (TNF)-*α* levels were delved by ELISA kits (Beyotime, Shanghai, China) as instructions. The values of OD_450 nm_ were probed by a microplate reader (Bio-Rad Labs, Richmond, CA, USA).

### 2.9. Measurement of Reactive Oxygen Species (ROS), Superoxide Dismutase (SOD), and Malondialdehyde (MDA)

ROS detection kit (Abcam, Shanghai, China) and SOD detection kit (Abcam) were leveraged to expose ROS and SOD levels in the supernatants of HUVECs, respectively, as protocols. The lipid peroxidation MDA assay kit (Abcam) was leveraged to detect the activity of MDA in HUVECs.

### 2.10. Flow Cytometry

Apoptosis assays were fulfilled by the Annexin V-Fluorescein Isothiocyanate (FITC)/Propidium Iodide (PI) Apoptosis Detection Kit (BD BiASciences, Franklin Lakes, NJ, USA). HUVECs were followingly treated with ox-LDL (100 *μ*g/mL) for 24 h. HUVECs were harvested, immersed twice in phosphate buffer saline (PBS), and specifically centrifuged at 1000 r/min for 5 min, with the supernatant removed. Then the HUVECs (1 × 10^6^ cells/mL) were subsequently resuspended in 100 *μ*L of binding buffer containing 5 *μ*L of Annexin V-FITC staining kit. After that, the HUVECs were accordingly incubated with 10 *μ*L PI for 15 min in darkness at ambient temperature. HUVECs were immersed in PBS, and the apoptotic rate was detected by flow cytometer (BD Biosciences, San Jose, CA, USA).

### 2.11. Dual-Luciferase Gene Reporter Assay

The full-length sequences of circ_0026218 and SIRT6 3′-UTR with wild-type (WT) or mutant (MUT) miR-338-3p binding sites were initially synthesized and subsequently cloned into psi-CHECK2 (Promega, Madison, WI, USA), and thus WT or MUT luciferase reporter vectors were generated (circ_0026218-WT and SIRT6-WT or circ_0026218-MUT and SIRT6-MUT). The luciferase reporter vector was subsequently cotransfected with miR-338-3p mimics or its negative control (miR-NC) in HEK-293 cells by Lipofectamine® 2000 (Invitrogen). 48 h later, the activity was recorded by the Dual-Luciferase Reporter Gene Assay Kit (Promega, Madison, WI, USA).

### 2.12. RNA Immunoprecipitation (RIP) Assay

RIP analysis was fulfilled by the Magna RIP RNA binding protein immunoprecipitation kit (Millipore, Bedford, MA, USA). 100 *μ*L of RIP lysis buffer, 0.5 *μ*L protease inhibitor mixture and 0.25 *μ*L RNase inhibitor were applied to prepare the complete RIP lysis buffer, which were mixed with HUVECs (5 × 10^5^ cells) for 1 h. Then the lysates were followingly incubated overnight at 4°C with magnetic beads coupled with argonaute2 (Ago2) antibody (Abcam) or immunoglobulin G (IgG) antibody (Abcam). The lysates were instantly treated with Proteinase K buffer, and the immunoprecipitated RNA was then extracted by the RNeasy MinElute purification kit (Qiagen, Shanghai, China), and circ_0026218 and miR-338-3p expressions in the immunoprecipitate were detected by qRT-PCR.

### 2.13. Western Blot Assay

HUVECs were initially lysed with RIPA lysis buffer (Beyotime) with phenylmethanesulfonyl fluoride (PMSF; Beyotime) and total cellular proteins were harvested. Protein extracts were collected and quantified by the BCA protein assay kit (BioRad, Hercules, CA, USA). Notably, equal amounts (30 *μ*g) of protein samples in each group were subjected to SDS-PAGE. Proteins were followingly transferred to PVDF membranes (Millipore), which were instantly blocked with 5% skimmed milk for 1 h and subsequently incubated with primary antibodies anti-SIRT6 (Abcam, ab191385, 1 : 1000) and anti-GADPH (Abcam, ab9485, 1 : 1000) at 4°C overnight. The membranes were then immersed in Tris-buffered saline with Tween-20 (TBST), incubated with secondary antibody (Abcam, ab97051, 1 : 5000) for 2 h and rinsed with TBST again. Ultimately, the protein signal was developed by the ECL Plus assay kit (Pierce, Rockford, IL, USA).

### 2.14. Statistical Analysis

Data were in the form of mean ± standard deviation. These data were tackled with SPSS 19.0 software (IBM Corp., Armonk, NY, USA). Discrepancies between two or among more groups were evaluated by Student's *t*-test or one-way ANOVA followed by Tukey's post-hoc test. *P* < 0.05 is of statistical significance.

## 3. Results

### 3.1. ox-LDL Inhibits circ_0026218 Expressions in HUVECs

**Figure 1:**
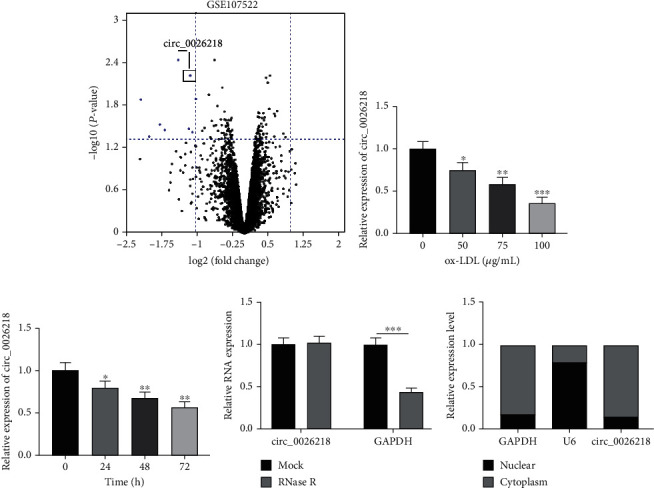
Downregulation of circ_0026218 expression in HUVECs with ox-LDL. (a) As the cutoff criteria (|log2 (*foldchange*)| >1 and *P* < 0.05), the volcano plot was used to analyze and screen out the circRNAs that were significantly up- and downregulated in the AS cell model induced by ox-LDL. Blue represents downregulated circRNAs, and black represents circRNAs that were not significantly differentially expressed. (b, c) circ_0026218 expression in HUVECs, stimulated with ox-LDL at discrepant concentrations (0 *μ*g/mL, 50 *μ*g/mlL, 75 *μ*g/mL, or 100 *μ*g/mL) and periods (0 h, 24 h, 48 h, and 72 h), were, respectively, probed by qRT-PCR. (d) circ_0026218 and GAPDH expressions, after the total RNA from HUVECs were incubated with RNase R, were exposed by qRT-PCR. (e) circ_0026218, U6, and GAPDH expressions in the nucleus and cytoplasm of HUVECs were subjected to qRT-PCR. ^∗^*P* < 0.05, ^∗∗^*P* < 0.01, ^∗∗∗^*P* < 0.001.

By tackling the microarray dataset GSE107522 in the public database, we found that circ_0026218 was downregulated in the *in vitro* model of ox-LDL-caused AS (Figure 1(a). To expound how circ_0026218 impacts the ox-LDL-initiated HUVECs, we probed into the impact of ox-LDL on circ_0026218 expressions in HUVECs under discrepant concentrations (0, 50, 75, and 100 *μ*g/mL) or different treatment times (0, 24, 48, and 72 h) by qRT-PCR, demonstrating that circ_0026218 expressions were gradually reduced concentration-dependently (Figure 1(b) and time-dependently (Figure 1(c). To verify the circularity of circ_0026218, we performed RNase R assay, which displayed that RNase R decreased the level of GAPDH mRNA, but circ_0026218 level was not significantly impacted (Figure 1(d). The analyses on the subcellular distribution of circ_0026218 displayed that circ_0026218 was mainly located in the cytoplasm of HUVECs (Figure 1(e), suggesting that circ_0026218 may interfere with the injury of endothelial cells and the development of AS.

### 3.2. Circ_0026218 Overexpression Enhances Viability, and Restrained Inflammatory Response, Oxidative Stress Damage and Apoptosis of HUVECs with ox-LDL Treatment

**Figure 2:**
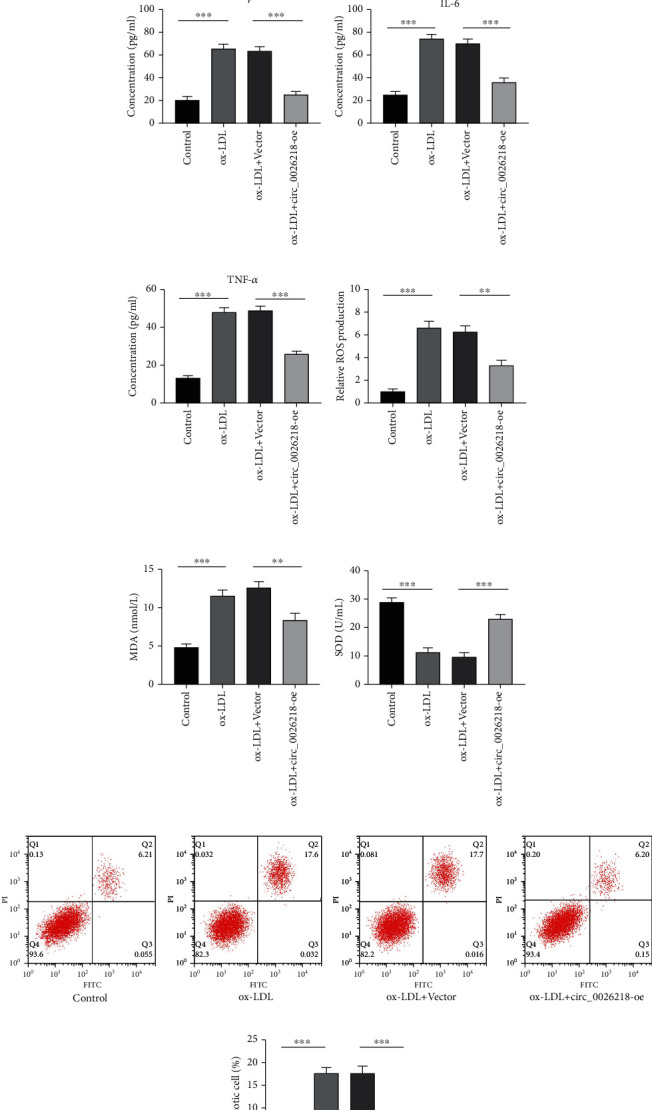
circ_0026218 overexpression enhances viability of HUVECs and inhibits inflammatory response, oxidative stress, and apoptosis. (a) circ_0026218 expressions in HUVECs transfected with circ_0026218 overexpression plasmid were examined by qRT-PCR. (b) The impact of circ_0026218 overexpression on the multiplication of HUVECs caused by ox-LDL was evaluated by the CCK-8 method. (c–e) The effects of circ_0026218 overexpression on IL-1*β*, IL-6, and TNF-*α* concentrations in ox-LDL-caused HUVECs were delved by ELISA. (f–h) The corresponding kits were employed to probe the contents of ROS, MDA, and SOD in HUVECs with ox-LDL after circ_0026218 overexpression. (i, j) Flow cytometry was applied for detecting ox-LDL-caused apoptosis of HUVECs after circ_0026218 overexpression. ^∗∗^*P* < 0.01, ^∗∗∗^*P* < 0.001.

To elaborate on the part of circ_0026218 in vascular endothelial cells, circ_0026218 overexpression vector (circ_0026218-oe) and control vector were meticulously transfected into HUVECs. We discovered that circ_0026218-oe transfection upregulated circ_0026218 expression in HUVECs (Figure 2(a). CCK-8 assay proved that ox-LDL blocked the growth of HUVECs and circ_0026218 overexpression could enhance the viability of HUVECs stimulated with ox-LDL (Figure 2(b). ELISA highlighted that ox-LDL inducement elevated IL-1*β*, IL-6, and TNF-*α* contents in HUVECs, while circ_0026218-oe transfection worked oppositely (Figures 2(c)–2(e). Moreover, ROS and MDA contents in the supernatant of HUVECs were greatly elevated and SOD level was demonstrably declined after ox-LDL stimulation, whereas circ_0026218 overexpression counteracted these effects (Figures 2(f)–2(h)). Furthermore, the apoptosis of HUVECs was mediated by ox-LDL inducement, which was reversed by circ_0026218 overexpression (Figures 2(i) and 2(j), indicating that circ_0026218 could play an inhibitory role in ox-LDL-caused injury of HUVECs.

### 3.3. Circ_0026218 Directly Targets miR-338-3p in HUVECs

**Figure 3:**
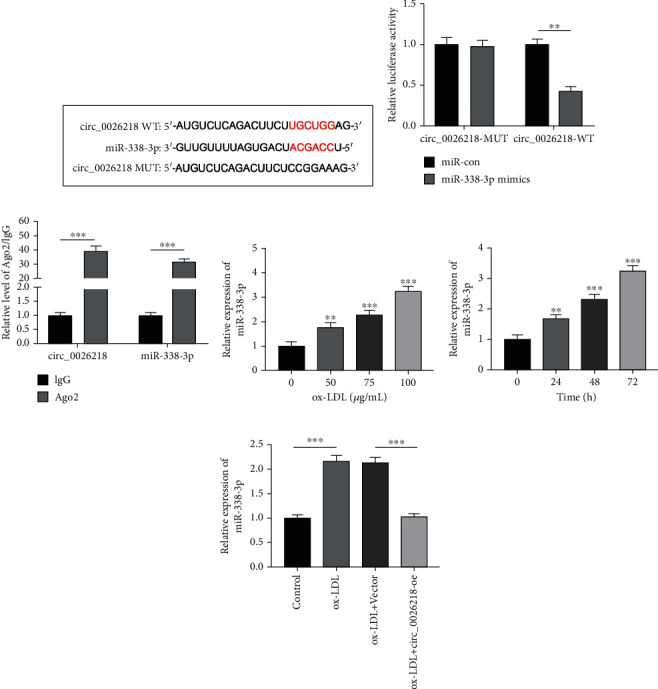
miR-338-3p is a target of circ_0026218. (a) The Circular RNA Interactome database was adopted to analyze the predicted binding sequence between miR-338-3p and circ_0026218. (b) This predicted binding was testified by dual-luciferase reporter gene assay. (c) The enrichment of circ_0026218 and miR-338-3p in HUVECs of anti-Ago2 group or the control group was exposed by RIP assay. (d) qRT-PCR was executed to probe miR-338-3p expressions in HUVECs with discrepant concentrations (0 *μ*g/mL, 50 *μ*g/mL, 75 *μ*g/mL, or 100 *μ*g/mL) of ox-LDL. (e) miR-338-3p expression in HUVECs treated with 100 *μ*g/mL ox-LDL for 0 h, 24 h, 48 h, and 72 h was probed by qRT-PCR. (f) qRT-PCR exposed miR-338-3p expressions in HUVECs after circ_0026218 overexpression. ^∗∗^*P* < 0.01, ^∗∗∗^*P* < 0.001.

CircRNAs usually sponges miRNAs to exert its biological effects [18]. To investigate the mechanism of circ_0026218 in ox-LDL-stimulated injury of endothelial cells, we predicted a hidden target miRNAs of circ_0026218 through the Circular RNA Interactome website, and found that there was a complementary binding site between circ_0026218 and miR-338-3p (Figure 3(a). As to the interplay of circ_0026218 and miR-338-3p, a dual-luciferase reporter gene assay proved that miR-338-3p overexpression dramatically attenuated the activity of circ_0026218-WT, but that of circ_0026218-MUT was not greatly influenced (Figure 3(b). RIP uncovered that circ_0026218 and miR-338-3p were remarkably enriched in anti-Ago2 group relative to anti-IgG group (Figure 3(c). qRT-PCR uncovered that ox-LDL treatment could promote miR-338-3p expressions in HUVECs concentration- and time-dependently (Figures 3(d) and 3(e). Notably, it was also observed that circ_0026218 overexpression in HUVECs repressed miR-338-3p expressions (Figure 3(f).

### 3.4. SIRT6 Is a Downstream Target of miR-338-3p

**Figure 4:**
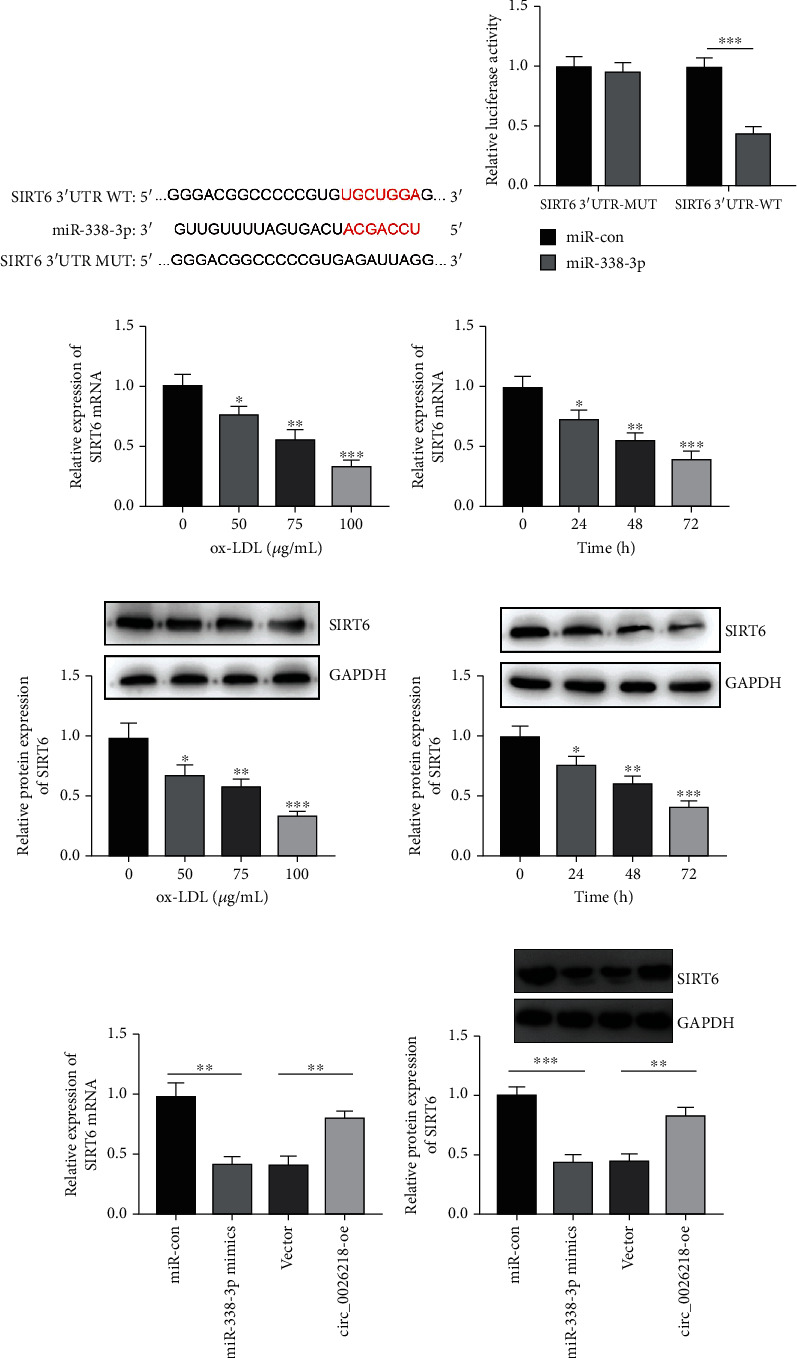
SIRT6 is a downstream target of miR-338-3p. (a) The TargetScan database was exploited to predict the binding sequence between miR-338-3p and SIRT6 3′-UTR. (b) The predicted binding relations were testified by dual-luciferase reporter gene assay. (c–f) SIRT6 mRNA expressions and protein levels in HUVECs treated with discrepant concentrations (0 *μ*g/mL, 50 *μ*g/mL, 75 *μ*g/mL, or 100 *μ*g/mL) of ox-LDL for 24 h or 100 *μ*g/mL ox-LDL for varied time points (0 h, 24 h, 48 h, or 72 h) were exposed by qRT-PCR and Western blot. (g, h) SIRT6 mRNA and protein expression in HUVECs with miR-338-3p or circ_0026218 overexpression were detected by qRT-PCR and Western blot. ^∗^*P* < 0.05, ^∗∗^*P* < 0.01, ^∗∗∗^*P* < 0.001.

To elucidate the promising mechanism of circ_0026218/miR-338-3p axis in modulating ox-LDL-caused injury of vascular endothelial cells, we predicted the hidden targets of miR-338-3p by TargetScan database and discovered that this miRNA had a complementary binding site with SIRT6 (Figure 4(a). As dual-luciferase gene reporter assay confirmed, miR-338-3p overexpression significantly inhibited the activity of SIRT6-WT, while that of SIRT6-MUT was not significantly influenced (Figure 4(b). qRT-PCR and Western blot displayed that ox-LDL restrained SIRT6 mRNA and protein expressions in HUVECs concentration- and time-dependently (Figures 4(c)–4(f)). In addition, miR-338-3p overexpression remarkably depressed SIRT6 mRNA and protein expressions in HUVECs as against those of the miR-con group (Figures 4(g) and 4(h); circ_0026218 overexpression increased SIRT6 mRNA and protein expressions in HUVECs as against the control group (Figures 4(g) and 4(h)).

### 3.5. circ_0026218 Upregulates SIRT6 Expression and Inhibits ox-LDL-Induced Functional Impairment in HUVECs via Targeting miR-338-3p

**Figure 5:**
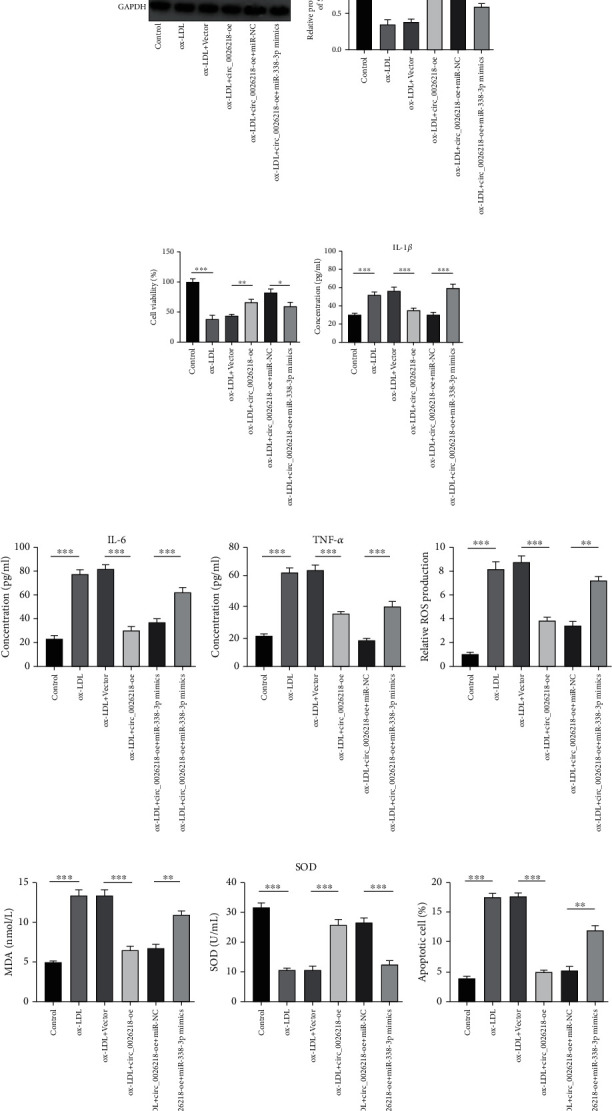
circ_0026218 inhibits ox-LDL-stimulated damage in HUVECs via targeting miR-338-3p. HUVECs were transfected with the circ_0026218 overexpression plasmid or/and miR-338-3p mimic, and treated with 100 *μ*g/mL ox-LDL for 24 h. HUVECs without any treatment served as controls. (a) miR-338-3p expressions were probed by qRT-PCR. (b, c) SIRT6 mRNA and protein expressions were exposed by qRT-PCR and Western blot. (d) The viability of HUVECs after transfection was probed by CCK-8 method. (e–g) IL-1*β*, IL-6, and TNF-*α* contents were delved by ELISA. (h–j) The corresponding kits exposed the levels of ROS, MDA, and SOD in HUVECs after transfection. (k–l) The apoptosis was probed by flow cytometry. ^∗^*P* < 0.05, ^∗∗^*P* < 0.01, ^∗∗∗^*P* < 0.001.

Rescue experiments were accomplished to further elucidate the interplay between circ_0026218 and miR-338-3p/SIRT6 axis in the injury of HUVECs. qRT-PCR uncovered that transfection of circ_0026218-oe restrained miR-338-3p expressions in HUVECs, while miR-338-3p overexpression reversed this effect (Figure 5(a). qRT-PCR and Western blot assays highlighted that circ_0026218-oe transfection promoted SIRT6 mRNA and protein expressions in HUVECs, while miR-338-3p overexpression rescued this effect (Figures 5(b) and 5(c). As CCK-8 assay highlighted, miR-338-3p mimics transfection could block the viability of HUVECs promoted by circ_0026218 overexpression (Figure 5(d). In addition, circ_0026218 overexpression inhibited ox-LDL-induced IL-1*β*, IL-6, TNF-*α*, ROS, and MDA production and elevated SOD level in HUVECs, and these impacts were reversed by miR-338-3p mimics (Figures 5(e)–5(j)). Cell apoptosis was remarkably decreased by upregulating circ_0026218 expressions, and this change was rescued by miR-338-3p mimics transfection (Figures 5(k) and 5(l). The above results implied that circ_0026218/miR-338-3p/SIRT6 axis is important for modulating the injury of HUVECs.

## 4. Discussion

Vascular endothelial cell dysfunction is a pivotal hallmark of AS development. Recently, circRNAs were reported to regulate the phenotype of endothelial cells. For example, circ_0065149 overexpression restrains ox-LDL-induced injury of HUVECs and suppresses inflammatory responses and apoptosis [9]. circ_0001445 overexpression reverses the mitigated viability of HUVECs caused by ox-LDL via activation of the SRSF1/*β*-catenin axis [20]. circ_0001445 overexpression restrains ox-LDL-initiated functional impairment and apoptosis in HUVECs [21]. This work focused on how circ_0026218 functions in HUVECs with ox-LDL. We proved that ox-LDL restrained circ_0026218 expressions in HUVECs concentration- and time-dependently. In addition, circ_0026218 overexpression enhanced the viability of HUVECs and inhibited inflammatory response, oxidative stress and apoptosis, suggesting that circ_0026218 may have an ameliorative effect on vascular endothelial cells and thus inhibit AS progression.

MiRNAs are promising candidates for the diagnosis and therapy of cardiovascular diseases [22]. Some studies have also covered vital roles of miRNAs in modulating the injury of vascular endothelial cells. For instance, miR-92a-3p accelerates ox-LDL-induced apoptosis of HUVECs via modulating the SIRT6/MAPK signaling pathway [23]. MiR-126-5p strengthens endothelial viability and limits AS via depressing DLK1 [24]. Reportedly, circRNAs can sponge miRNA to regulate the biological behavior of cells [25]. Additionally, the circRNA-miRNA-mRNA axis has been widely reported to interfere with the pathogenesis of AS [26, 27]. To elucidate the regulating mechanism of circ_0026218 in ox-LDL-stimulated injury of HUVECs, we predicted the target miRNAs of circ_0026218 by bioinformatics analysis and discovered that miR-338-3p could be decoyed by circ_0026218. The function of miR-338-3p has been covered in cancer biology in many previous studies. For instance, miR-338-3p is lowly expressed in prostate cancer [28], liver cancer [29], and colorectal cancer [30] and it inhibits tumor progression. MiR-338-3p is elevated in the aortic tissues of rat model with AS and it facilitates AS development [31]. This work proved that miR-338-3p was abundantly expressed in HUVECs treated with ox-LDL, which was coherent to the findings of the previous work [15]. Furthermore, miR-338-3p overexpression rescued the impacts of circ_0026218 upregulation on HUVECs, indicating that circ_0026218 could ameliorate ox-LDL-mediated injury of HUVECs via targeting miR-338-3p.

SIRT6 is a highly conserved NAD (^+^)-dependent deacetylase involved in DNA damage repair, telomere stabilization, glucose and lipid metabolism, and oxidative stress [32]. Reportedly, SIRT6 features prominently in cardiovascular disease [33]. Specifically, SIRT6 enhances angiogenesis and hemorrhage of carotid plaque via modulating HIF-1*α* and reactive oxygen species [34]. SIRT6 reduces the generation of foam cells by prompting autophagy and cholesterol efflux under ox-LDL condition [35]. This study found that SIRT6 was lowly expressed in HUVECs with ox-LDL, which was coherent to the preceding study [23]. In addition, SIRT6 could be a downstream target of miR-338-3p in HUVECs. We found that circ_0026218 could upregulate SIRT6 expression in HUVECs by adsorbing miR-338-3p, showing that circ_0026218/miR-338-3p/SIRT6 regulatory network may be involved in AS progression.

But there are certain limitations. First, we only used the ox-LDL-induced injury model of HUVECs for our study. We did not assess whether circ_0026218 could modulate the injury of HUVECs induced by other factors; secondly, we only concentrated on the protective effect of circ_0026218 *in vitro*. Animal models should be considered in following studies to validate our findings; finally, other targets of circ_0026218 need to be expounded.

On all accounts, circ_0026218 expressions were inhibited in HUVECs treated with ox-LDL. Mechanistically, circ_0026218 upregulated SIRT6 via targeting miR-338-3p, thus promoting the viability and inhibiting the inflammatory response, oxidative stress, and apoptosis of HUVECs. This work reveals new potential molecular mechanisms in AS progression and provides an emerging theoretical basis for treating AS.

## Data Availability

The data used to support the findings of this study are available from the corresponding author upon request.
